# Disordered eating and the muscular ideal

**DOI:** 10.1186/2050-2974-1-15

**Published:** 2013-04-25

**Authors:** Scott Griffiths, Stuart B Murray, Stephen Touyz

**Affiliations:** 1 School of Psychology, University of Sydney, Sydney, NSW, Australia; 2 Redleaf Practice, 5 Redleaf Ave, Wahroonga, Sydney, NSW, Australia

**Keywords:** Disordered eating, Muscularity, Male body image, Muscular ideal

## 

Despite sparse scientific attention, research efforts during the past decade have begun to reveal the complexity of male body image concerns. Early research conceptualised and measured male body dissatisfaction in terms of the drive for thinness and excess body fat E.g. [1]. However, most men in Western societies desire a body that combines low body fat with well-developed musculature, and which is further characterised by broad shoulders, a narrow waist, and big biceps [2,3]. The simultaneous pursuit of overt musculature and low body fat has led researchers to conceptualise muscle dissatisfaction and body fat dissatisfaction as separate but related constructs that are equally central to male body image [4]. Recent research has been dedicated to the development of instruments that measure fat- and muscularity-related body image concerns in men, so that the full spectrum of male body dissatisfaction can be assessed [5,6].

However, in contrast to research efforts on body image pathology, little research has attempted to delineate various dimensions of disordered eating as they pertain to the muscular ideal. Current conceptualisations of disordered eating are synonymous with body fat, fat loss, weight loss and calorie restriction, all of which characterise the drive for thinness, not the drive for muscularity. This bias in our understanding of what constitutes disordered eating is evident in the research instruments used to measure disordered eating behaviours. The gold standard Eating Disorders Examination [7] contains mostly thinness-oriented items. For example, “Have you gone for periods of eight or more waking hours without eating anything in order to influence your shape or weight?”, “Have you had a strong desire to lose weight” and “Have you wanted your stomach to be empty?” Such items, whilst appropriate for measuring thinness-oriented eating pathology, are insensitive to eating pathology motivated by the desire for greater muscularity.

The pursuit of muscularity can be characterised by a variety of rule-driven eating behaviours, many of which are antonymic to eating behaviours that are motivated by the desire to lose body fat and become thin. Muscularity-oriented disordered eating behaviours include very high levels of protein consumption, severe restriction of non-protein related dietary components, interrupting important activities to accommodate frequent eating, continued food consumption despite feeling full, eating very frequently (every 2 to 3 hours), liquefying or blending food for easier intake, consuming a large proportion of calories in liquid form, compensatory restriction of carbohydrates or fats due to deviation from one’s training regime, and the use of appearance enhancing drugs such as steroids, “testosterone boosters” and other supplements [8,9]. Contemporary conceptualisations of disordered eating do not easily accommodate these muscularity-oriented facets of disordered eating.

The insensitivity of the current conceptualisation of disordered eating may explain why muscle dissatisfaction and the drive for muscularity have not been reliably associated with disordered eating. Studies on straight and gay men have found that body fat dissatisfaction robustly predicts disordered eating, but have found no such association with muscle dissatisfaction [10-13]. Furthermore, perceived pressure to lose weight directly predicts disordered eating amongst French adolescent boys, but not pressure to gain muscle [14]. Little research has examined the association between drive for muscularity and disordered eating amongst women. Rather than conclude that the desire to be bigger and more muscular does not entail disordered eating, the absence of positive findings may be a consequence of measures that simplistically equate disordered eating with fat loss, weight loss and calorie restriction. Instruments that are sensitive to the full spectrum of disordered eating are needed to enable future investigations into whether muscularity-oriented disordered eating is associated with distress and disability in the same way as thinness-oriented disordered eating.

To improve our understanding of disordered eating behaviours, particularly in men, a reconceptualisation of disordered eating may be warranted; one which focuses on the rules that underlie disordered eating, rather than the specific direction of the eating behaviour. For example, consider an individual who desires thinness and eats very infrequently, and another individual who desires increased muscularity and eats often. The underlying rule that makes this behaviour disordered in both individuals is that they eat meals based on time intervals rather than based on feeling hungry. It is apparent that multiple body ideals, from very thin to very muscular, motivate disordered eating. However, the current conceptualisation of disordered eating is concerned only with thinness, weight loss and calorie restriction, making it outdated and in need of reform.

### Competing interests

The authors declare that they have no competing interests.

### Authors’ contributions

SG conceived the idea and drafted the manuscript. SM and ST edited the manuscript and made important revisions. All authors read and approved the final manuscript.
